# Learning-related brain hemispheric dominance in sleeping songbirds

**DOI:** 10.1038/srep09041

**Published:** 2015-03-12

**Authors:** Sanne Moorman, Sharon M. H. Gobes, Ferdinand C. van de Kamp, Matthijs A. Zandbergen, Johan J. Bolhuis

**Affiliations:** 1 Cognitive Neurobiology and Helmholtz Institute, Departments of Psychology and Biology, Utrecht University, Utrecht, The Netherlands; 2 Department of Biology, Boston University, Boston, MA, USA; 3 Neuroscience Program, Wellesley College, Wellesley, MA, USA

## Abstract

There are striking behavioural and neural parallels between the acquisition of speech in humans and song learning in songbirds. In humans, language-related brain activation is mostly lateralised to the left hemisphere. During language acquisition in humans, brain hemispheric lateralisation develops as language proficiency increases. Sleep is important for the formation of long-term memory, in humans as well as in other animals, including songbirds. Here, we measured neuronal activation (as the expression pattern of the immediate early gene *ZENK*) during sleep in juvenile zebra finch males that were still learning their songs from a tutor. We found that during sleep, there was learning-dependent lateralisation of spontaneous neuronal activation in the caudomedial nidopallium (NCM), a secondary auditory brain region that is involved in tutor song memory, while there was right hemisphere dominance of neuronal activation in HVC (used as a proper name), a premotor nucleus that is involved in song production and sensorimotor learning. Specifically, in the NCM, birds that imitated their tutors well were left dominant, while poor imitators were right dominant, similar to language-proficiency related lateralisation in humans. Given the avian-human parallels, lateralised neural activation during sleep may also be important for speech and language acquisition in human infants.

Humans and songbirds share the ability of vocal production learning by imitation – a trait that is absent in our closest relatives, the apes^1^,^2^,^3^. There are remarkable behavioural, neural and genetic similarities between speech acquisition in human infants and birdsong learning^1^,^2^,^4^,^5^,^6^. In humans, there is a language-related brain network involving multiple functional circuits. It is well established that Broca's area in the left frontal lobe and Wernicke's area in the left temporal lobe are central brain regions in this network^7^ (Fig. 1a). Among other functions, Broca's area is involved in speech production and generation and processing of language syntax, while Wernicke's area plays a major role in speech perception and comprehension^7^,^8^. In the songbird brain, there is a similar neural dissociation, with a neural network for song production (the ‘song system', including the song nucleus HVC, used as a proper noun)^9^,^10^,^11^ and brain regions that are involved in song perception and recognition (including the caudomedial nidopallium, NCM)^12^,^13^,^14^. Thus in a functional sense, these networks are considered to be analogous to Broca's and Wernicke's areas in humans, respectively^1^,^15^. Neural activation in relation to speech and language in Broca's and Wernicke's areas occurs predominantly in the left hemisphere in human babies, infants and adults^7^,^16^,^17^,^18^,^19^,^20^,^21^. There is growing evidence for lateralisation of neural activation in relation to song production and perception in songbirds as well; although it is not yet clear which specific roles the hemispheres play^22^,^23^.

Sleep plays an important role in learning and memory in mammals and insects, particularly in the process of consolidation^24^,^25^,^26^,^27^. Consolidation refers to a process in which short-term memories are transformed into more stable and long-term stored memory representations^24^. It has been suggested that memory consolidation may also occur during sleep, to prevent interference between daily activities and memory consolidation^24^.

Songbirds, including zebra finches, have a sleep pattern that is remarkably similar to that of humans, with a similar distribution of sleep phases^28^,^29^. Sleep is important for memory consolidation in birds (reviewed in Refs. 28, 30). Jackson and colleagues^31^ showed that a period of sleep after visual imprinting training is necessary for consolidation to occur in domestic chicks. Similarly, it was shown that sleep enhances consolidation of auditory memory in European starlings^32^,^33^. Furthermore, sleep in juvenile zebra finches affects tutor song imitation^34^, and learning-related changes in neuronal activity during sleep have been observed in a song system nucleus (the robust nucleus of the arcopallium, or RA) after a single day of song tutoring in juvenile zebra finches^35^.

Here, we investigated brain lateralisation during sleep in songbirds. Previously, we found that there is memory-related neuronal activation in the NCM of juvenile zebra finches during sleep^36^. More recently, we demonstrated memory-specific left-hemispheric dominance of neuronal activation in the NCM and general left-hemispheric dominance in HVC in awake juvenile zebra finches^37^. In human infants, brain lateralisation during language processing is dependent on language proficiency^20^. Here we show that there is a similar pattern of learning-dependent brain lateralisation in songbirds.

## Results

**Figure 1:**
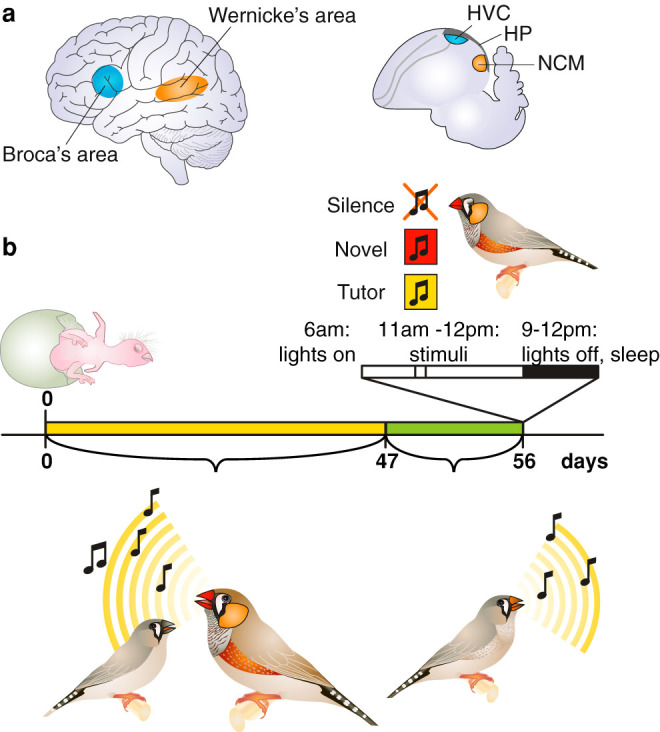
Schematic of experimental design: Immediate Early Gene (IEG) expression was measured bilaterally in juvenile male zebra finches during sleep. (a) We measured IEG expression in brain areas NCM, HVC and hippocampus. The NCM is part of an auditory pathway that could be considered analogous to Wernicke's area in the human brain. HVC is part of a song production pathway that could be considered analogous to Broca's area in the human brain. (b) Birds were reared with their parents until 47 days after hatching. During this time, they could memorize and imitate their fathers' songs. Then, they were moved to siblings-only groups to prevent them from hearing their fathers' songs. Two nights before the experiment, juveniles were moved to sound-isolation chambers. On the day of the experiment, one group of experimental subjects did not receive auditory stimulation (‘silence'), a second group was exposed to unfamiliar conspecific song (‘novel'), and a third to their fathers' song (‘tutor'). All groups consisted of 6 animals. They were undisturbed during the rest of the day. During the subsequent night, when the birds were asleep, they were sacrificed.

Figure 1b depicts the experimental design. After tutoring, we separated the juveniles from their parents, so the juveniles could not hear their tutor's song anymore. When the juveniles were approximately 56 days old and in the sensorimotor learning phase, we exposed the juveniles to either the song of their father, which they had started to imitate, to an unfamiliar conspecific song or to silence. We hypothesized that playbacks of the target song (tutor song) during the day would trigger memory consolidation, which might occur during sleep. Thus, instead of measuring neuronal activation caused by stimulus exposure directly, we measured neuronal activation during sleep (Fig. 1b). Birds were sacrificed 3 hours after the lights were turned off. Video analysis indicated that the mean time that the juveniles had slept before perfusion was 2 hours and 32 minutes (range: 1 hour and 56 minutes–2 hours and 51 minutes).

**Figure 2:**
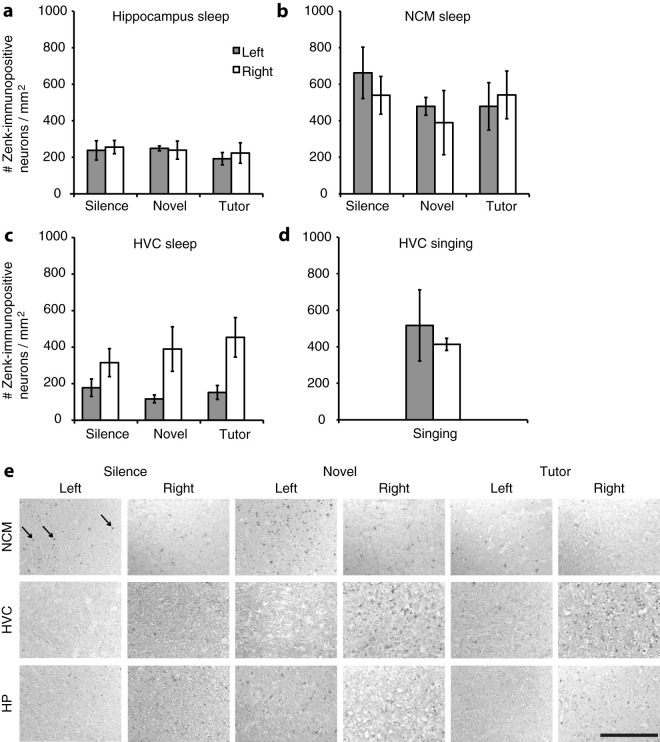
Neuronal activation in HVC is lateralised during sleep. Mean number of Zenk-immunopositive neurons per square millimetre is shown for the different brain regions. Grey bars represent the left hemisphere, and white bars represent the right hemisphere. Error bars represent ± s.e.m. (a) In the hippocampus, a control brain region that is not implicated in song learning, Zenk expression levels are low and not lateralised. (b) In the NCM during sleep, there were high levels of Zenk expression but no differences between groups or hemispheres. (c) We found high activation levels in the right HVC in all conditions, while Zenk expression levels were low in the left HVC. (d) In a separate group of juvenile males in which IEG expression was measured immediately after they had been singing, there were high levels of Zenk expression in both left and right HVC. (n = 4) (e) Photomicrographs of juvenile zebra finch brains showing Zenk-immunostaining. Representative images at the level of the lateral NCM, HVC, and hippocampus (HP) are shown for the silence, novel, and tutor stimulus groups, in the left and right hemispheres. Arrows in the upper left image indicate examples of zenk-positive cells (black immunolabelling). Scale bar represents 0.2 mm.

We quantified the number of Zenk-immunopositive neurons bilaterally in the NCM, HVC and hippocampus (Fig. 1a) to investigate lateralisation of neuronal activation during sleep (Fig. 2a, b, c). An overall repeated measures ANOVA revealed significant effects of Brain Region (F_2,9_ = 17.501, p = 0.001) and Hemisphere (F_1,10_ = 5.485, p = 0.041), and a significant interaction between these two factors (F_2,9_ = 4.411, p = 0.046). Therefore, we conducted subsequent analyses on the three brain regions separately. In the hippocampus, the level of activation was low and did not differ between stimulus groups (F_2,14_ = 0.439, p = 0.653, n.s.) or hemispheres (F_1,14_ = 0.107, p = 0.748, n.s.; Fig. 2a).

### Learning-related lateralisation of neuronal activation in the lateral NCM

**Figure 3:**
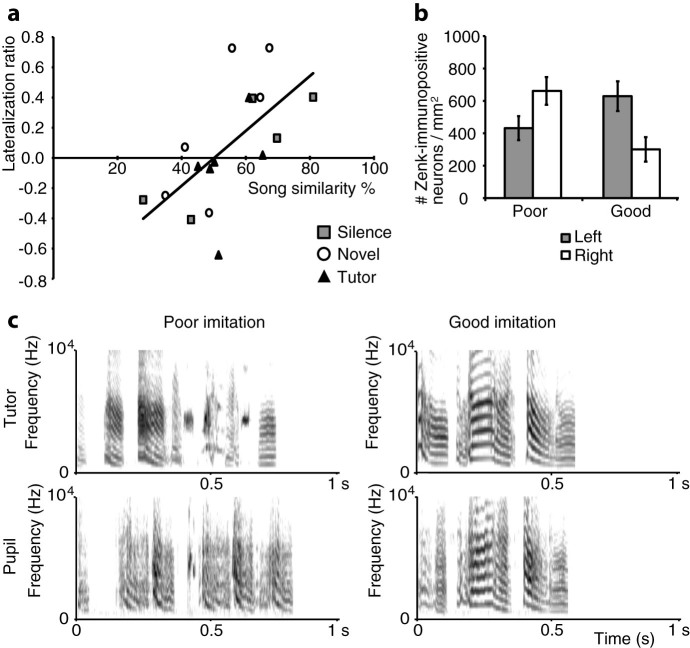
Neuronal activation in the lateral NCM is lateralised during sleep: poor learners show right-dominant activation in the NCM, while good learners have a left-dominant expression pattern. (a) Lateralisation ratios (L − R)/(L + R) were calculated for each subject from the number of Zenk-immunopositive cells per square millimetre in the NCM. Song similarity percentages on the x-axis indicate to which degree the juveniles had imitated their fathers' songs. Black triangles represent birds that were exposed to tutor song during the day; open circles, novel song; grey squares, silence. The regression line shown is for the three stimulus groups together, and the correlation is significant (Pearson's r = 0.630, p = 0.007). (b) Mean number of Zenk-immunopositive neurons per square millimetre in the NCM for all experimental groups grouped together (silence, novel, tutor), but individuals are divided into two groups based on learning proficiency. ‘Poor': song similarity <55% ( = mid-range); ‘good': song similarity >55%. Grey bars represent the left hemisphere, and white bars represent the right hemisphere. Poor, n = 9, good, n = 8. Error bars represent ± s.e.m. (c) Spectrograms of juveniles that produced a poor or good imitation of their tutor's song. Left: Juvenile had a song similarity of 28% with its tutor. Right: Juvenile had a song similarity of 81% with its tutor.

We expected to find differential neuronal activation between the tutor song-exposed juveniles, and the ones that heard novel song or remained in silence during the day. However, there was no significant difference in Zenk expression between the stimulus groups (silence, novel and tutor) or between the left and right NCM (Fig. 2b). Figure 3a shows lateralisation ratios (see methods) in the NCM during sleep, for the juvenile birds in all three stimulus groups, in relation to song similarity scores. A multiple regression analysis showed that the lateralisation ratio could be predicted by the factors Song similarity and Stimulus groups (F(2,14) = 4.715, p = 0.027). Moreover, Song similarity statistically significantly predicted the level of lateralisation (t = 3.006, p = 0.009), while Stimulus group did not (t = −0.364, p = 0.721). Also the interaction between Song similarity and Stimulus groups did not predict the lateralisation ratio (F(1,16) = 0.475, p = 0.501). When the results of the stimulus groups were analysed together, there was a significant positive correlation between song similarity of the juveniles and the lateralisation ratio in the NCM during sleep (Pearson's r = 0.630, p = 0.007, n = 17; Fig. 3a). Thus, in contrast to stimulus-specific lateralisation during the day, where we previously found a correlation in the tutor stimulus-group only^37^, here we found a significant correlation in all stimulus groups. These results show that there is left hemisphere dominance regarding neuronal activation in the lateral NCM during sleep, proportional to the strength of song learning that has occurred.

The results shown in figure 3a suggest that lateralisation is dependent on the degree of song learning. There was substantial between-subject variability in the mean song similarity percentage between each of the juveniles and their tutors. Since there were no significant differences in Zenk expression in the NCM or mean song similarity with the tutor song between the three stimulus groups, we separated the birds into two learning proficiency groups: ‘poor learners' and ‘good learners' (see example sonograms in figure 3C). We set the boundary between poor learners and good learners at the middle of the song similarity range, at 55%. The mean song similarity of poor learners with their tutors was 43.44% (n = 9), while of good learners it was 65.83% (n = 8). The difference in song similarity between the poor and good learners could have been caused by more variation from song to song in the poor learners, thus a less stable song than the good learners because they are slower with song learning, or alternatively, by an actual qualitative difference in their vocal learning abilities. We calculated the song similarity between multiple song renditions of the juveniles (on average 25 per bird) and the tutor song. Then, we calculated the mean and variance of the song similarity scores of each juvenile. The mean standard deviation of the poor learners was 11.96, and of the good learners 12.01. Therefore, there was no significant difference between the poor and good learners in the variation between song similarities (One-way ANOVA: F_1,16_ = 0.000, p = 0.984), suggesting that the difference between poor and good learners was not due to differences in song stability.

When we analysed the Zenk expression of poor learners and good learners separately, there was differential lateralisation. Repeated measures ANOVA revealed a significant interaction between Hemisphere (Left or Right NCM) and Learning proficiency (Poor or Good; F_1,15_ = 11.615, p = 0.004). Poor learners were right-dominant (t = −3.299, p = 0.011, n = 9), while good learners showed left dominance in the NCM (t = 4.219, p = 0.004, n = 8; Fig. 3b).

### Neuronal activation in HVC is right dominant during sleep

A repeated-measures ANOVA revealed right-dominance of neuronal activation in HVC during sleep in all stimulus groups (effect of Hemisphere, F_2,11_ = 12.209, p = 0.005; Fig. 2c). Multiple regression analysis showed that neither left- nor right-sided activation in HVC, nor the lateralisation ratio in HVC, could be predicted by the factors Song similarity, Stimulus group or Amount of singing (all p > 0.05).

In contrast, in an earlier study with awake juvenile zebra finch males we found left-dominance in HVC^37^. There are two important differences between these two studies. First, in the previous study neuronal activation was determined during daytime right after stimulus exposure (the lights were off for four hours to prevent the birds from singing, before and during stimulus exposure), while here, we assessed neuronal activation when the birds were asleep at nighttime (the lights were off for three hours prior to sacrifice). Second, the birds in the previous study sang in the first two hours of the day only, and did not sing in the 4 hours before they were sacrificed, while in the current study, the birds could sing during the whole day, before the lights were turned off in the evening. To investigate which of these factors may have affected the difference in HVC lateralisation between day and night, we conducted a study with a separate group of juvenile male zebra finches (reared in the same way as the experimental males in the main experiment) that sang during the day, and were also sacrificed during the day (n = 4). In accordance with the literature (reviewed in Ref. 23), we found that the left and right HVC were activated approximately equally in these birds (t = 0.545, p = 0.623, n = 4; Fig. 2d).

## Discussion

We demonstrated differential lateralisation of neuronal activation in a ‘Wernicke-like' brain region (the NCM) and a ‘Broca-like' brain region (HVC) during sleep in juvenile male zebra finches. The strength of song learning was correlated with the lateralisation ratio in the NCM during sleep, with greater song similarity related to stronger neuronal activation in the left hemisphere. When we divided the experimental subjects into “poor learners” and “good learners”, we found that for the NCM, the right hemisphere was predominantly activated during sleep in poor learners, while the left hemisphere was dominant in good learners. HVC activation of sleeping juveniles was right-dominant independent of the strength of song learning.

The neuronal activation we found during sleep could be spontaneous (i.e. due to molecular re-activation or replay of neuronal activity), or, alternatively, it could be the result of sustained activation from events happening during the day (i.e. the bird hearing its own song before sleep). The latter possibility is unlikely for two reasons. First, nuclei labeled for zenk can be observed as early as 15 minutes after start of stimulation, and zenk levels peak 1–2 hours after stimulus onset. Zenk expression reduces almost back to baseline 4 hours after stimulation^38^. Thus, it is likely that the neurons that were immunopositive had been activated during sleep. Second, activation patterns detected during the day are almost a mirror image of the patterns we reported here during sleep.

**Figure 4:**
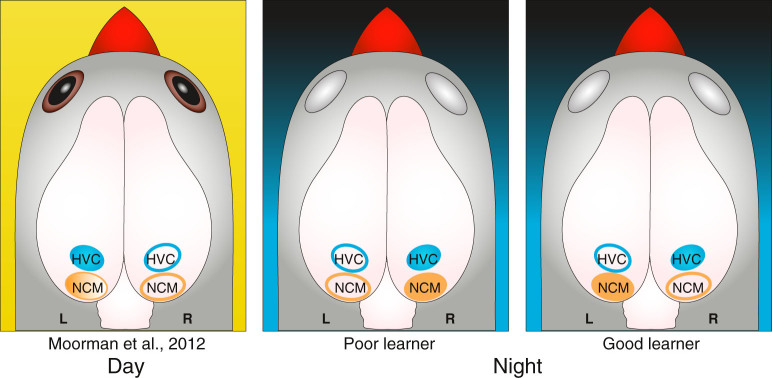
Results overview. Left panel: Previously in a similar experiment, we found that HVC showed a left-dominant activation in awake and silent juvenile zebra finch males, while the NCM was left-dominantly activated during tutor song playback only (and not during silence or novel, demonstrating memory-specific lateralisation)^37^. Middle and right panel: Here we have demonstrated that during sleep at night, HVC was activated right-dominantly in all groups of birds, while the NCM showed differential lateralisation between learning proficiency groups. In poor learners, NCM activation was right-dominant (middle panel), while in good learners NCM activation was left-dominant (right panel).

Previously we demonstrated that in awake juvenile zebra finches, neuronal activation in the NCM and HVC is also lateralised, but in a different manner: the left NCM of awake juveniles is dominant when the bird is exposed to tutor song, while HVC of awake, non-singing zebra finches is left dominant irrespective of the stimulus to which they are exposed^37^. Taken together these results suggest differential lateralisation of neuronal activation depending on the behavioural state of the animal (sleeping or awake; Fig. 4).

The finding that poor learners exhibited neuronal activation predominantly in the right NCM, while neuronal activation in good learners was left-lateralised, is reminiscent of language-related lateralisation in humans that are acquiring a new language. Language-related neuronal activation in babies and infants is already left-dominant (i.e., Refs. 16, 18, 39,40,41). In children and adults who are developing their language skills, e.g., acquiring language or learning a second language, brain regions activated during speech perception were right-dominant or bilateral and encompassing relatively large areas. Activity shifted to the left side of the brain or was reduced in extent with increased language proficiency, with left-dominance at higher ages and more developed language abilities (as reviewed in e.g., Refs. 23, 42). Except for one case report^43^, as far as we are aware, no studies in humans have investigated language-related lateralisation during sleep.

What is the function of brain lateralisation? In humans, different brain regions are involved in different aspects of language processing, for example neural activation during processing of prosody is found predominantly in the right temporal lobe^7^. One hemisphere might be better-suited for a specific task than the other, and it has been suggested that the left-sided auditory cortex is more efficient in processing speech-like temporal patterns^44^. Likewise, it has been suggested that there is a functional division of the two hemispheres in songbirds. In starlings, song processing in the left hemisphere might be more focussed on individual recognition of birds far away, while the right hemisphere may be specialised for analysing long and complex song sequences that birds in close proximity sing^45^. Another hypothesis is that lateralisation improves dual-task performance (i.e., when two tasks are executed simultaneously; Refs. 23, 46). Language lateralisation seems to be beneficial for performance; there are clinical examples of lateralisation abnormalities associated with language impairments such as dyslexia^47^. Aberrant lateralisation might also affect language performance in autism^48^ and schizophrenia^49^.

When different results on lateralisation in songbirds are compared^23^, there are differences between studies, probably caused by the songbird species that was tested, the type of stimulus used, the methods employed and whether the birds were awake, anesthetised or asleep. Generally, both left and right HVC contribute to singing in zebra finches, in an alternating way (“rapid hemispheric switching”^50^, bilateral contributions that alternate on a millisecond time scale; see Ref. 23), while in canaries the left HVC is crucial for singing (e.g., Ref. 51, see also Ref. 23). During song perception, in HVC and the NCM usually one hemisphere is selective for some song types over others (e.g. bird's own song vs conspecific song or conspecific vs heterospecific song), and the other hemisphere responds more to birdsong in general, although the direction of lateralisation is not consistent. This directional ambiguity could be due to the behavioural state of the animal (awake, sleeping or under anesthesia, see Ref. 23). It has been argued that anesthesia induces “artificial sleep”^52^,^53^,^54^,^55^. Most studies measuring neural activity during anesthesia or sleep show a right-dominant pattern of neuronal activation, while studies in awake animals mostly show left-sided dominance, in both the NCM and HVC (reviewed in Ref. 23). Our findings concerning behavioural state-dependent neuronal activation in HVC fit this pattern of results.

In addition to the NCM, HVC might play a role in tutor song memory^11^,^56^,^57^. Song memory acquisition might involve dynamic interaction between the NCM and HVC, perhaps to provide a tutor song template with which to compare the birds' own^1^,^14^,^58^. The left NCM could be involved in tutor song memory processing, while the right NCM may subserve reciprocal interaction between song recognition memory and motor learning systems, including HVC. Although a direct anatomical connection between the NCM and HVC is not known, the NCM projects to the CM (caudal mesopallium)^59^, and the CM to HVC^60^. Furthermore, data from an experiment combining electrophysiology with retrodialysis suggest that there is a functional connection between the NCM and HVC. Inhibition of estrogen-synthesis in the NCM decreased selectivity for birds' own song in HVC neurons, while increasing the levels of neuroestrogens in the NCM increased song selectivity in HVC^61^.

Hemispheric specialisation with a unilateral temporal memory representation was found in filial imprinting in domestic chicks, where the left IMM (intermediate and medial mesopallium) has been shown to be a permanent memory store, while the right IMM is implicated in the formation of a temporal memory store elsewhere in the brain (the hypothetical S′)^62^,^63^. In juvenile zebra finches, the right NCM could have a similar role to the right IMM of chicks, being involved in the formation of a representation of bird's own song in the song system. In that case, the more the birds already learned from their tutor's song, the less neural interaction is needed and the less activation we would find in the right NCM.

Alternatively, the left NCM could inhibit activation in the right NCM in good learners. In humans, it is hypothesized that the left temporal lobe inhibits activation in its right counterpart and when this does not happen very efficiently, for example in schizophrenic patients, right temporal lobe activation can cause the illusion of hearing voices^49^. Interestingly, most of the IMM neurons of chicks activated after imprinting contain GABA^63^, and similarly, almost half of the neurons that are Zenk-immunopositive after song stimulation in the NCM are GABA-ergic cells^64^, indicating that inhibition does play an important role in the NCM. Since we do not know of any direct projections between the left and right NCM, if the left NCM would indeed inhibit activation in the right NCM, it should project via a longer route, for example via L3 (a primary auditory region) to RA cup (the surrounding region of the robust nucleus of the arcopallium) to Ov (nucleus ovoidalis), and cross hemispheres at the level of the midbrain or the thalamus^5^,^59^,^65^. In accordance with both hypotheses, the most extreme left-dominant birds (which were the best learners) had very low levels of activation in the right NCM in comparison to right-dominant birds.

In conclusion, we found lateralised brain activation during sleep in juvenile songbirds that were in the process of learning their song. A ‘Broca-like' brain region (HVC) showed differential activation of the two hemispheres depending on behavioural state: left-dominant during the day^37^, bilateral during singing, and right-dominant during sleep. Spontaneous neuronal activation during sleep in a ‘Wernicke-like' brain region (the NCM) was left-lateralised in good learners, and right-lateralised in poor learners. Given the parallels that exist between birdsong and human speech, lateralised neuronal activation during sleep may also be important for speech and language acquisition in human infants, and lateralisation might be a fundamental process for auditory-vocal learning.

## Methods

### Animals

The 22 juvenile zebra finch males were bred at the central animal facility of Utrecht University. The subjects have been used in a previous study^36^ for analysis of the left hemisphere. Here, we took new photomicrographs of the left NCM and HVC of the same subjects, and added photomicrographs of the right NCM and right HVC and left and right hippocampus of the same birds. Thus, we conducted entirely new measurements of neuronal activation in the left and right hemispheres of these subjects, and calculated relative activation ratios. Experimental procedures were in accordance with European law and approved by the Animal Experiments Committee of Utrecht University.

### Experimental design

Birds were kept in breeding cages with their parents and siblings until 47 days post-hatching (dph). At 47 dph, all males from a clutch were removed from the breeding cages and kept in communal cages, and moved to individual cages at least 48 hours before the experiment. The experiments were performed at a mean age of 56 dph (range: 54–59 dph). Subjects were exposed to their tutor's song, novel conspecific song, or silence. After that, the birds were not disturbed until sacrifice at midnight, 3 hours after the lights were turned off. Video and sound recordings were made throughout the experimental day, to record the birds' singing activity, the total daytime napping duration and to measure how long the birds had been asleep before being sacrificed.

### Tissue collection and immunocytochemistry

At midnight, the birds were deeply anesthetised with an overdose of Natriumpentobarbital (Nembutal, Ceva Sante Animale, Libourne, France, 0.06 mL i.m.) and perfused with phosphate-buffered saline (PBS), followed by fixation with fresh, ice-cold 4% paraformaldehyde in PBS. Brains were dissected out, postfixed at 4°C, cryoprotected in 10%, 20% and 30% sucrose in PBS at 4°C, frozen and kept at −20°C until cut. Parasagittal 20-μm sections were made on a cryostat and mounted on poly-L-lysine coated slides. The brains were incubated with primary polyclonal rabbit antiserum for egr-1 (Zenk; Santa Cruz Biotechnology; dilution 1:1000), overnight at 4°C; biotinylated goat antirabbit (IgG, Vector Laboratories, Burlingame, CA; dilution 1:100), for 1 hour at room temperature; ABC (avidin-biotinylated enzyme complex, Vector Elite Kit, Vector Laboratories), for 1 hour at room temperature; and diaminobenzidine (DAB) medium containing 0.034% H_2_O_2_ for 6 minutes at room temperature. Zenk, the acronym of zif-268, egr-1, ngf-Ia and krox-24, is used as a marker for neuronal activation and is expressed upon postsynaptic membrane depolarization^38^.

### Image analysis

Quantification of Zenk-immunopositive cells was performed for the lateral and medial NCM, HVC and hippocampus as described before^66^. Brain areas were identified using a stereotaxic atlas that is available online^67^. Digital photographs were taken using a Leica DFC 4206 camera and the Leica Application Suite program on an Axioskop (Zeiss, Germany) with 20× objective. Image analysis was performed with a personal computer-based system using KS400 version 3.0 software (Zeiss, Oberkochen, Germany). A program had been previously developed in KS400 to quantify the number of immunoreactive cells semi-automatically^66^. Counts of zenk-immunopositive cells per square mm were obtained in three sections for each brain region in both hemispheres per animal. These were averaged for further statistical analysis. All image analysis was performed blind as to the experimental history of the subject.

### Behavioural analysis

We analysed video data to quantify the time a bird had been asleep during the day and subsequent night prior to sacrifice as described previously^36^. Sound recordings were analysed using Sound Analysis Pro to calculate song similarities between the subjects and their tutors. To do so, five representative motifs of the juveniles were randomly selected from the afternoon on the day of the experiment, between 12 and 4 PM. Tutor songs were recorded between 1–12 months before the experiment. All songs were high-pass filtered and the root-mean-square amplitude was equalized using Praat software^68^. We calculated the ‘percentage similarity', which is a measurement of syllable copying, with the computer program Sound Analysis Pro [http://soundanalysispro.com/]. Based on multiple features (Wiener entropy, spectral continuity, pitch and frequency modulation), this comparison provides an objective quantification of song similarity^69^.

### Statistical analyses

We conducted repeated measures ANOVA to compare the effect of stimulus exposure on the Zenk response in the left and right hemisphere per brain region. We further analysed the brain regions separately using repeated measures ANOVA. To test for lateralisation effects between the learning proficiency groups, a repeated-measures ANOVA was performed. To test for lateralisation effects within the learning proficiency groups, post hoc paired t-tests were performed. A lateralisation ratio was calculated by dividing the difference in Zenk expression levels that we counted in the left- and right-sided regions by the total of those: (left − right)/(left + right). This lateralisation ratio enabled us to look at true lateralisation levels not influenced by differences in absolute neuronal activation^37^. A multiple regression analysis was run to predict lateralisation levels from song similarity data and stimulus exposure groups. Data were analysed using SPSS 20.0.0 (IBM Corporation).

## Author Contributions

S.M., M.A.Z. and J.J.B. conceived and designed the current study. S.M., S.M.H.G., F.C.K., M.A.Z. and J.J.B. executed the current study. S.M.H.G. conceived and designed, and S.M.H.G., M.A.Z. and J.J.B. executed the original sleep study. S.M., S.M.H.G., M.A.Z. and J.J.B. analysed the data and S.M., S.M.H.G. and J.J.B. wrote the paper.
